# Butyrate reduces adherent-invasive *E. coli*-evoked disruption of epithelial mitochondrial morphology and barrier function: involvement of free fatty acid receptor 3

**DOI:** 10.1080/19490976.2023.2281011

**Published:** 2023-12-11

**Authors:** Samira A. Hamed, Armaan Mohan, Saranya Navaneetha Krishnan, Arthur Wang, Marija Drikic, Nicole L. Prince, Ian A. Lewis, Jane Shearer, Åsa V. Keita, Johan D. Söderholm, Timothy E. Shutt, Derek M. McKay

**Affiliations:** a Gastrointestinal Research Group, Inflammation Research Network, Host-Parasite Interactions Program, Department of Physiology & Pharmacology, Calvin, Phoebe and Joan Snyder Institute for Chronic Diseases, Cumming School of Medicine, University of Calgary, Calgary, Canada; b Departments of Medical Genetics and Biochemistry & Molecular Biology, Alberta Children’s Hospital Research Institute, Hotchkiss Brain Institute, Snyder Institute, Cumming School of Medicine, University of Calgary, Calgary, Canada; c Calgary Metabolomics Research Facility, Department of Biological Sciences, Faculty of Science, University of Calgary, Calgary, Canada; d Department of Biochemistry and Molecular Biology, Faculty of Kinesiology, University of Calgary, Calgary, Canada; e Department of Biomedical and Clinical Sciences, Division of Surgery, Orthopedics and Oncology, Linköping University, Linköping, Sweden

**Keywords:** Pathobiont, mitochondrial dynamics, gut epithelium, organoid, T84 cells, permeability

## Abstract

Gut bacteria provide benefits to the host and have been implicated in inflammatory bowel disease (IBD), where adherent-invasive *E. coli* (AIEC) pathobionts (*e.g*., strain LF82) are associated with Crohn’s disease. *E. coli*-LF82 causes fragmentation of the epithelial mitochondrial network, leading to increased epithelial permeability. We hypothesized that butyrate would limit the epithelial mitochondrial disruption caused by *E. coli*-LF82. Human colonic organoids and the T84 epithelial cell line infected with *E. coli*-LF82 (MOI = 100, 4 h) showed a significant increase in mitochondrial network fission that was reduced by butyrate (10 mM) co-treatment. Butyrate reduced the loss of mitochondrial membrane potential caused by *E. coli*-LF82 and increased expression of PGC-1α mRNA, the master regulator of mitochondrial biogenesis. Metabolomics revealed that butyrate significantly altered *E. coli*-LF82 central carbon metabolism leading to diminished glucose uptake and increased succinate secretion. Correlating with preservation of mitochondrial network form/function, butyrate reduced *E. coli*-LF82 transcytosis across T84-cell monolayers. The use of the G-protein inhibitor, pertussis toxin, implicated GPCR signaling as critical to the effect of butyrate, and the free fatty acid receptor three (FFAR3, GPR41) agonist, AR420626, reproduced butyrate’s effect in terms of ameliorating the loss of barrier function and reducing the mitochondrial fragmentation observed in *E. coli*-LF82 infected T84-cells and organoids. These data indicate that butyrate helps maintain epithelial mitochondrial form/function when challenged by *E. coli*-LF82 and that this occurs, at least in part, via FFAR3. Thus, loss of butyrate-producing bacteria in IBD in the context of pathobionts would contribute to loss of epithelial mitochondrial and barrier functions that could evoke disease and/or exaggerate a low-grade inflammation.

## Introduction

As omnipresent companions, commensal bacteria are an important determinant of health and susceptibility to disease, via direct interaction between bacteria and the host, and the liberation of metabolites. This is best illustrated in the gut, where the complexity of the ecosystem therein is being unraveled, and the implications for perturbations in the bacterial community for enteric homeostasis and immunity, and the impact on distant organs, such as the brain, are being defined.^1^ Microbiota-derived molecules hold the promise of a pharmacopoeia^2^ and the importance of bacteria-derived short chain fatty acids (SCFA) to health is supported by numerous studies.^3^ For example, butyrate is recognized as the major energy source for the colonic epithelium, can direct the development of regulatory immune cells, and can exert anti-inflammatory effects via G-protein receptor signaling and gene regulation (*e.g*., via inhibition of histone deacetylase activity).^3,4^

Commensal gut bacteria confer many benefits on their host: conversely, they have been implicated as triggers in the pathogenesis of the idiopathic inflammatory bowel diseases (IBD), Crohn’s disease and ulcerative colitis.^5^ Adherent-invasive *E. coli* (AIEC) have emerged as putative pathobionts in IBD, being more prevalent in patient-biopsies compared to those from healthy individuals. They have the ability to survive in macrophages and to trigger a pro-inflammatory response in these cells and in epithelial cells, and to exaggerate disease in murine gut inflammation.^6–11^

Since Roediger’s insight that IBD might be a metabolic disorder due to mitochondrial dysfunction,^12^ data have accumulated from observations in tissues from individuals with IBD, rodent models of colitis, and *in vitro* models to support the hypothesis that epithelial mitochondrial damage contributes to the onset or exacerbation of IBD, in part via loss of control of epithelial barrier function.^13–16^ Several microbial pathogens cause mitochondrial damage and disrupt the balance of fission and fusion of the mitochondrial network that is critical to effective function.^17,18^ Recently, we reported that the prototypic AIEC pathobiont isolated from a patient with Crohn’s disease, *E. coli*-LF82, evoked substantial fragmentation of the mitochondrial network in the human colon-derived T84 epithelial cell line.^19^ The mitochondrial fragmentation observed at early stages of infection (*i.e.*, 4–6 h) was reduced by treatment with the drugs P110 or M-divi1 that inhibit dynamin-related protein-1 (Drp1) mediated fission, while the later stages of infection (*i.e.*, 8–12 h) were associated with loss of the mitochondrial pro-fusion factor, optic atrophy factor-1 (Opa1). The epithelial mitochondrial fragmentation was dependent on viable *E. coli*-LF82 capable of binding to and invading the enterocyte and was not observed with the spent medium from *E. coli*-LF82 cultures.^19^

Juxtaposing these findings, it was hypothesized that under normal circumstances the commensal bacteria limit the ability of pathobionts to cause epithelial mitochondrial damage and increase gut permeability. Butyrate, which is produced by bacterial fermentation and has known immunomodulatory and metabolic functions,^4^ was used to test this hypothesis in a proof-of-concept paradigm. While butyrate affected neither the growth of *E. coli*-LF82 nor its ability to invade epithelia, when added as a simultaneous co-treatment with *E. coli*-LF82, it significantly preserved the epithelial mitochondrial network via maintenance of mitochondrial membrane potential, and signaling through free fatty acid receptor (FFAR)-3. Based on the dysbiosis that accompanies IBD, and that often involves loss of SCFA-producing species of bacteria,^20^ we speculate that the local loss of butyrate contributes to host susceptibility to pathobionts capable of disrupting epithelial mitochondrial activity, resulting in reduced epithelial barrier function and promoting enteric inflammation.

## Materials & methods

### Cell culture and drug treatments

Colonic biopsies from healthy controls (individuals undergoing colon cancer screening) and patients with Crohn’s disease (identified as coming from an inflamed or non-inflamed region of the patients’ colon as determined by the attending gastroenterologist (Suppl. Table S1)) were provided via the Intestinal Inflammation Tissue Bank (Univ. Calgary) for qPCR analysis under ethics protocol REB-15-0586. The Human Organoid Innovation Hub (HOIH) at the Univ. of Calgary provided human colonic organoids (three donors) (ethics approval REB-18-0104). The enterocytes were isolated, cultured and differentiated following a published protocol.^21^

The human colonic T84 (male; passages 52–100) and CaCo2 (male; passages 100–120) epithelial cell lines were used. T84-epithelia were cultured in 1:1 Dulbecco’s Modified Eagle’s Medium (DMEM)/F12 Ham medium (Sigma Chemical Co., St. Loius, MO) containing HEPES (2 mM, Sigma), L-glutamine (2.68 mM, Gibco, ThermoFisher Scientific Inc., Waltham, MA), sodium pyruvate (0.6 mM, Sigma), sodium bicarbonate (0.015%, Gibco), and penicillin-streptomycin (120 U/mL penicillin, 0.12 mg/mL streptomycin, Sigma), and 10% fetal bovine serum (FBS; pH 7.4; ThermoFisher).^19^

Commensal *E. coli* (strain HB101) was maintained on Luria Bertani (LB) agar (VWR International, Mississauga, ON) and grown in LB broth (Becton Dickinson Canada Inc., Mississauga, ON).^22^ Adherent invasive *E. coli* (AIEC) strain LF82 was maintained on Columbia sheep blood agar and cultured in antibiotic-broth (#70184; Fluka Analytical, Sigma).^7^ Bacterial growth curve analysis was performed as previously described.^22^ For co-culture experiments, 10^6^ epithelial cells were seeded on sterile coverslips in 12-well plates and grown to ~70% confluence as judged by phase-contrast light microscopy and were then treated with 10^8^ colony forming units (cfu)/mL of bacteria for a multiplicity of infection (MOI) of ~100.^19^ When fewer epithelial cells were used (*e.g*., in 96-well plates), numbers of bacteria were adjusted to maintain MOI = 100.

The following reagents were used: the short chain fatty acids, sodium acetate (3 mM), sodium propionate (3 mM) and sodium butyrate (3, 10, 20 mM; all Cayman Chemicals, Ann Arbor, MI)^23^ the long-chain fatty acid, palmitate (50 μM): Palmitoly-l-carnitine (1 mM was toxic on T84 cells; Cayman Chemical); the Gi subunit inhibitor pertussis toxin (50 ng/mL; TOCRIS Bioscience, Bristol, UK)^24^ the FFAR2 antagonist (S)-3-(2-(3-Chlorophenyl) acetamido)-4-(4-(trifluoromethyl) phenyl) butanoic acid (CATPB) (10 μM; Sigma). The FFAR3 antagonist/hydroxcarboxylic acid receptor-2 (HCAR) agonist, β-hydroxybutyrate (5 mM; Sigma),^25^ the histone deacetylase (HDAC) inhibitor, trichostatin A (2 μM; Sigma),^26^ the fatty acid oxidation inhibitor, etomoxir (10 μM; Sigma),^27^ the metabolic toxin protonophore carbonyl cyanide 4-(trifluoromethoxy) phenylhydrazone (FCCP) (10 μM; Sigma)^19^ and, the FFAR3 agonist, AR420626 (25 μM; Sigma)^28^ The doses of drugs used were tested and were neither bactericidal nor bacteriostatic in this study as determined by bacterial growth curve analyses.^22^

### *Bacterial growth and epithelial invasion by* E. coli*-LF82*

*E. coli*-LF82 (10^8^ cfu) was incubated in antibiotic-free cell culture medium ± sodium butyrate (10 or 20 mM) and grown at 37°C for 4–24 h and bacterial growth assessed by measuring optical density as described before.^19^

Following T84 epithelial (10^6^ cells) and *E.* *coli*-LF82 (10^8^ cfu) co-culture in 12-well plates, a 10 μL aliquot of medium was taken for bacterial culture, the culture media was then aspirated and replaced with 1 mL medium containing gentamycin (200 μg/mL; Sigma) for 1.5 h to kill extracellular bacteria. The gentamycin-containing medium was removed, the epithelial cells lysed with 0.1% Triton-X 100/PBS, and the extracts serially diluted and cultured at 37°C for 18 h and cfu counted. Data are presented as % invasion.^19^

### Metabolomic analyses

The methods used for metabolomics^29^ sample preparation, high resolution liquid chromatography mass spectrometry (LC-MS) data collection, and statistical analysis have been described in detail elsewhere.^30,31^ Briefly, extracellular metabolites from cell cultures were harvested by collecting and centrifuging (10,000 *g*, 10 min) the cell media to remove cells. Next, 10 μL of the resulting supernatants were extracted in 190 μL of 50% methanol/water (v/v).^29^ All metabolite extracts were separated using hydrophilic interaction liquid chromatography (HILIC; Thermo Syncronis 2.1 mm x 100 mm x 1.7 µm column) and reverse-phase ion pairing chromatography (RPIP; Zorbax SB-C18, 2.1 mm x 50 mm x 1.8 μm; Agilent Canada, Mississauga, ON) on Vanquish chromatography system, and acquired in negative mode ionization on Q-Exactive Orbitrap Mass Spectrometer (ThermoFisher) following published methods.^32^ LC-MS data were analyzed using the El-MAVEN software package.^33^ Metabolites were identified by matching the observed m/z signals (±10 ppm) and chromatographic retention times to those observed from commercial metabolite standards (LSMLS^TM^, Sigma). Statistical analysis was performed by FUGO-MS (www.LewisResearchGroup/software) and GraphPad Prism 9.

### ATP and IL-8 measurement

Epithelial intracellular levels of ATP were measured using the Cell Titer-Glo Luminescent Cell Viability Assay (Promega, WI, USA) and applied according to the manufacturer’s instructions, with luciferase activity read in a Victor3V 1420 Multi-label Counter (PerkinElmer, Waltham, MA). Briefly, 5 × 10^5^ T84 cells were seeded into 12-well plates, and 48 h later were treated with 5 × 10^7^ cfu *E. coli*-LF82 (MOI = 100) ± butyrate (10 or 20 mM). Four hours later, cell extracts were prepared and ATP levels were determined from a standard curve and normalized to sample protein content, measured by the Bradford assay (Bio-Rad Laboratories, Mississauga, ON).^19^ IL-8 was measured by ELISA using paired antibodies from R&D Systems.

### Mitochondrial network quantification

T84 (or CaCo2) epithelial cells were seeded in 8-well chamber slides (Thermo Fisher) or sterile coverslips, cultured for 48 h and then treated with Mitotracker Red CMXROS probe (100 nM; Molecular Probes, Eugene, OR) diluted in Hanks Balanced Salt Solution (HBSS) for 35 min at 37°C. After washing, cells were stained with the nuclear dye Hoechst (1 mg/mL, Thermo Fisher). Bacteria (adjusted to MOI = 100) ± the SCFA or drug treatment (some drugs were used as a pre-treatment, see figure legends) were added for 4 h prior to live cell imaging on an Olympus spinning disk confocal microscope (SD-OSR) with the x100 objective (microscope was set at 180 ms for the SD-561 red laser and 150 ms for SD-405 blue laser to detect Mitotracker red and Hoechst signal, respectively). Mitochondrial morphology was investigated in a semi-quantitative manner as previously described.^19^ Blinding was achieved by randomly choosing cells based on Hoechst-stained nuclei and then switching the fluorescent channel to image Mitotracker. Mitochondrial networks were rated as fused (mostly interconnected networks), fragmented (majority of mitochondria are spherical) or intermediate. Twenty separate fields of view spanning the epithelial monolayer were selected. A complementary quantitative analysis was performed on captured images using an ImageJ software kindly proved by J. Mewburn (Queens University, Kingston, Ontario, Canada).^34^ Background signals are removed and the mitochondrial network skeletonized followed by counting and measurement of the particles in the designated area. Fused mitochondrial networks are larger in size with a smaller particle count than fragmented networks. Thus, mitochondrial fragment count (MFC) is equal to the particle count divided by the total observation area.

Human colonic organoids provided by the HOIH (Univ. Calgary) were grown on 3-μm filter supports (Corning, ThermoFisher) or 8-well chamber slides.^21^ Colonic organoids were imaged live with Mitotracker or by immunostaining for translocase of outer mitochondrial membrane (TOMM-20) on fixed cells.^35^ After treatment (*i.e*., exposure to *E. coli*-LF82 ± butyrate or AR420626 for 4 h), cells were fixed in 4% paraformaldehyde (15 min, 37°C), followed by three rinses in PBS and permeabilized in 0.2% triton-X 100 (15 min, room temperature (RT)). Following rinsing and blockade with 10% goat serum in PBS for 1 h at RT, rabbit-anti-human primary TOMM-20 antibody (ab186735; 1:100 diluted in 5% goat serum; Abcam, Waltham, MA) was added for 24 h at 4°C, then rinsed and secondary goat-anti-rabbit antibody was applied (A11011, Alex Fluor 568, 1:1,000; ThermoFisher) for 2 h at RT. Cells were then rinsed, stained with 4’,6-diamidino-2-phenylindole (DAPI: 1;1,000, 10 min RT), rinsed and mounted in Dako fluorescent medium (Dako North America Inc., Agilent). Images were captured on an Olympus spinning disk confocal microscope (x100 objective) and analyzed as described for Mitotracker staining.

### Measurement of mitochondrial membrane potential

T84 epithelial cells were seeded into 6-well plates (1×10^6^/well) cultured for 48 h and following the experimental treatment, cells were stained with tetramethyl rhodamine-ethyl ester (TMRE: 400 nM, 40 min, 37°C; ThermoFisher) dye for mitochondrial membrane potential. FCCP (10 μM) was used as a positive control for loss of mitochondrial membrane potential. Cells were then washed with PBS, dissociated with accutase (Sigma), and 1 mL PBS (37°C) was added to each well followed by gentle pipetting to increase dissociation. Cells were pelleted and re-suspended in 700 μL phenol red-free culture medium, passed through a 100 µm cell strainer (#22363549; ThermoFisher) and transferred to polystyrene 5 mL flow tubes (Falcon), and stained with DAPI(5 min). prior to assessment. A Becton-Dickson FACS CANTO cytometer supported by the BD FACS Diva software (BD Biosciences, Franklin Lakes, NJ) was used to measure fluorescence signal intensity from the dyes. The TMRE signal was detected by a PE laser (561-red) and the DAPI signal was detected by a BV421 laser (405-violet). The DAPI signal was used to exclude dead cells and mitochondrial membrane potential was measured as percent of change in mean fluorescence intensity (MFI) of TMRE compared to control cells, where lower MFI indicates increased mitochondrial membrane depolarization. Data were analyzed using Flow-Jo analysis software.^36^

### Measurement of mRNA

RNA extraction from T84 cells and colonic biopsies was accomplished using the Aurum Total RNA Mini Kit (Bio-Rad; Mississauga, ON), with the resultant quality and quantity of RNA assessed in a ND-1000 Nano Drop Spectrophotometer (ThermoFisher). cDNA synthesis was performed using an I-Script kit (Bio-Rad). PCR primers were designed and purchased from Invitrogen (ThermoFisher) and validated for qPCR in melt-curve analyses. The qPCR reaction was performed on a Step One Plus Real-Time PCR system using the IQ-SYBER Green Super mix (Bio-Rad) with 0.5 µg of cDNA and the primers (sequences are listed in Suppl. Table S2).^37^ The resulting CT values were normalized to the 18s RNA house-keeping gene or RPL27 for mt-DNA^38^ quantification by the ΔΔCT method and compared to control cells.

### Immunoblotting

Following the experimental treatment, protein was extracted from human organoids and epithelial cell lines and immunoblotting performed as previously described^19^ using mouse anti-human FFAR3 (1:1,000; #66811–1-1 g, Proteintech, Rosemount, IL) and GAPDH (1:1,000; ab8245, Abcam) antibodies, and a goat anti-mouse HRP-conjugated secondary antibody (1:5,000; sc-2031; Santa Cruz Biotech., Pasa Robles, CA).

### Data presentation and analysis

Data are presented as mean ± standard error of the mean (SEM) and ‘n’ is defined as the number of epithelial monolayers from a specified number of experiments. Statistical tests were performed in GraphPad Prism software version 9.3.1. For parametric data either a one-way or two-way ANOVA followed by Tukey’s multiple comparison posttest was performed, while normalized data (i.e. flow cytometry and qPCR) were analyzed by the non-parametric Kruskal–Wallis test followed by Dunn’s multiple comparison test. A statistically significant difference was accepted at *p* <0.05.

## Results

### E. coli*-induced epithelial mitochondrial fragmentation is reduced by co-treatment with butyrate*

**Figure 1.**
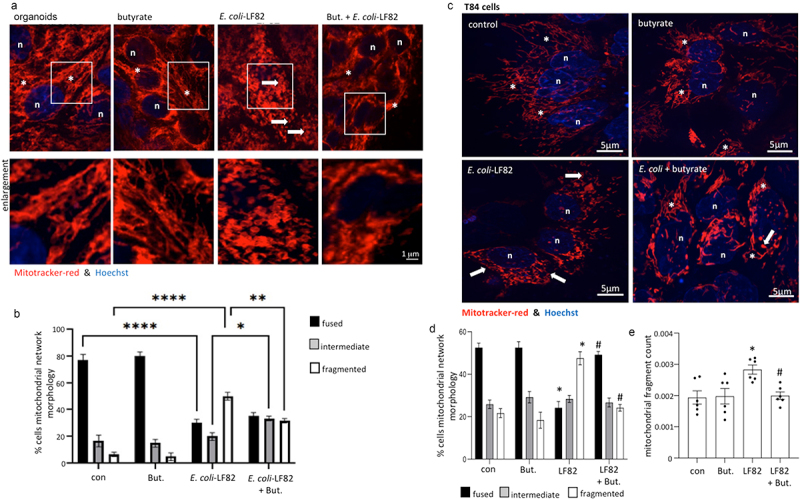
*E. coli*-LF82 evoked mitochondrial fragmentation of enteric epithelia is reduced in butyrate co-treated cells. Human colon organoids (a, b) or monolayers of the human colon-derived T84 epithelial cell line (c-e) were treated with *E. coli*-LF82 (10^8^ cfu, 4 h) ± co-treatment with sodium butyrate (But., 10 mM). Representative images were collected in a random fashion by first identifying epithelia nuclei (blue, n) and then swapping the confocal laser channel to assess the mitochondrial network (Mitotracker (red)). Twenty cells per monolayer were characterized by semi-quantitative analysis (b, d) and an image-J analysis program (e) (data are mean ± SEM, from 3 organoids and 6–9 epithelial monolayers assessed in 2–3 separate experiments; *, **, **** statistically different at *p* < .05, *p* < .01 and *p* < .001 respectively (panel b); * and #, *p* < .05 compared to control uninfected cells (con) and *E. coli*-LF82 only infected cells, respectively (panels d, e) by two-way ANOVA followed by Tukey’s multiple comparisons test; image *, fused mitochondrial network with elongated strands; arrow, fragmented, vesiculated area of the mitochondrial network).

Human colon-derived epithelial organoids exposed to *E. coli*-LF82 demonstrated significant mitochondrial fragmentation (Figure 1a), validating and extending previous findings with human epithelial cell lines^19^ (control bacteria were not included because we previously showed^19^ that neither *E. coli*-HB101 nor *E. coli*-F18 consistently caused epithelial mitochondrial fragmentation in time- and MOI-matched comparisons with *E. coli*-LF82 (Suppl. Fig. S1)). Reasoning that in healthy individuals butyrate is present in the gut lumen, organoids co-treated with butyrate and *E. coli*-LF82 displayed a less fragmented mitochondrial network compared to *E. coli*-LF82 only treated epithelia, with a significant increase in the percentage of cells classified as intermediate and fewer with fragmented mitochondrial networks as defined by semi-quantitative assessment (Figure 1a, b). Similar findings with butyrate were observed in *E. coli*-LF82-infected T84 epithelial cells (Figure 1c–e), supporting use of this model in mechanistic studies (and in the CaCo2 epithelial cell line (Suppl. Fig. S2)). While butyrate has been repeatedly implicated as important in intestinal homeostasis, many other short- and long-chain fatty acids (LCFA) are abundant in the gut.^39^ The SCFAs, propionate and acetate also antagonized the effect of *E. coli*-LF82-induced mitochondrial fragmentation in T84 cells and the mechanism of this requires further investigation and comparison with the effect of butyrate. In contrast, co-treatment with the LCFA, palmitate, did not affect *E. coli*-LF82-evoked disruption of the T84-epithelial mitochondrial network (Suppl. Fig. S3).

In accordance with this finding, a qPCR assessment of inflamed or non-inflamed colonic biopsies from individuals with Crohn’s disease revealed altered expression of genes associated with mitochondrial dynamics compared to controls; specifically increased mRNA expression of *DNM1L*, *MFN1* and *OPA1* (Suppl. Fig. S1). While this supports perturbed mitochondrial dynamics in IBD,^15^ we have no evidence of AIEC in the biopsies used here.

### *Butyrate is not cytotoxic or cytostatic on* E. coli

**Figure 2.**
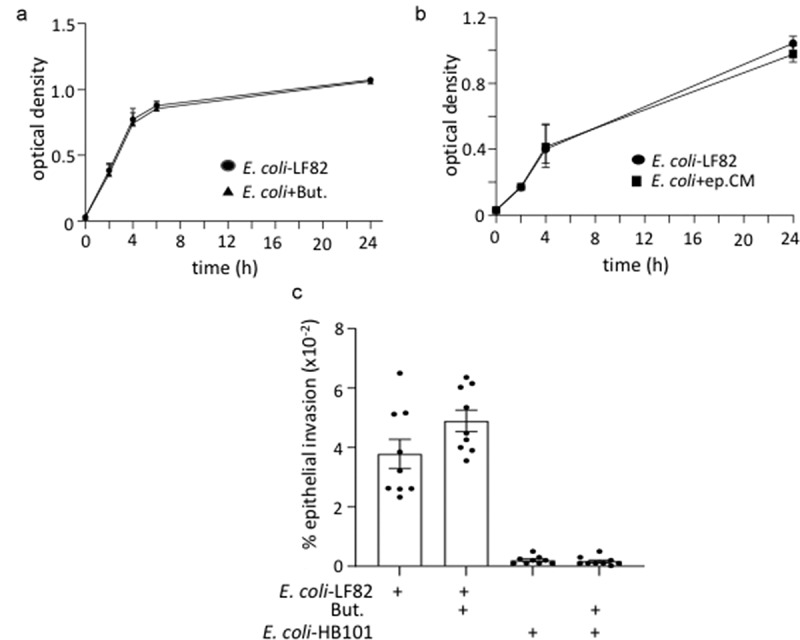
Butyrate does not kill *E. coli*-LF82. *E. coli*-LF82 (10^8^ cfu) only (no epithelium) were cultured in antibiotic-free medium with butyrate (But., 10 mM) (a) or a 50% spent-medium from T84+butyrate+*E. coli*-LF82 cultures (4 h, 0.2 μm filtered (ep.CM)) (b) and optical density measured over a 24 h period (*n* = 3). (c) Invasion assays revealed that butyrate did not alter the ability of *E. coli*-LF82 to invade T84 epithelia (multiplicity of infection = 100, 4 h; non-invasive *E. coli*-HB101 (10^8^ cfu) shown for comparison) (data are mean ± SEM, 9 epithelial monolayers from 3 experiments).

Butyrate suppression of *E. coli*-LF82 growth, or attachment and invasion of epithelial cells could limit the bacteria’s ability to evoke epithelial mitochondrial fragmentation. However, butyrate did not affect the growth of *E. coli*-LF82 (Figure 2a); neither did conditioned-medium from *E. coli*-LF82-infected+butyrate-treated epithelial cells (Figure 2b precluding the possibility that the enterocyte produced a bactericidal or bacteriostatic molecule), nor did butyrate affect *E. coli*-LF82 invasion of T84 epithelia (Figure 2c).

### *Butyrate modulates* E. coli *metabolism*

The metabolomics methods used here report on the extracellular metabolites that were consumed or secreted over a 4 h period by *E. coli*-LF82. As expected,^30^ the *E. coli* consumed carbohydrates and organic acid (e.g. glucose and pyruvate), amino acids (e.g. glutamine), and nucleotide precursors (e.g. hypoxanthine) and secreted a range of waste products downstream of each of these pathways (e.g. succinate, lactate, and xanthosine (Suppl. Fig. S5A)). Importantly, glucose levels were not depleted over the 4 h incubation indicating that the bacteria had access to their primary preferred carbon source throughout the experiment. These expected metabolic phenotypes were significantly altered when butyrate was added to *E. coli* cultures, resulting in diminished glucose uptake, elevated pyruvate catabolism, diminished lactate and succinate production, but elevated 3-hydroxybutyrate secretion (Suppl. Fig. S5B,C). In sum, metabolomics experiments showed that butyrate exposure directly affects the abundance of microbe-derived succinate and other metabolites that are directly linked to mitochondrial metabolism. These metabolic changes or butyrate-induced gene regulation in the *E. coli* may contribute to diminished epithelial mitochondrial fragmentation in the co-culture setting.

### *Butyrate in combination with* E. coli*-LF82 boosts epithelial IL-8 and PGC-1α mRNA expression*

**Figure 3.**
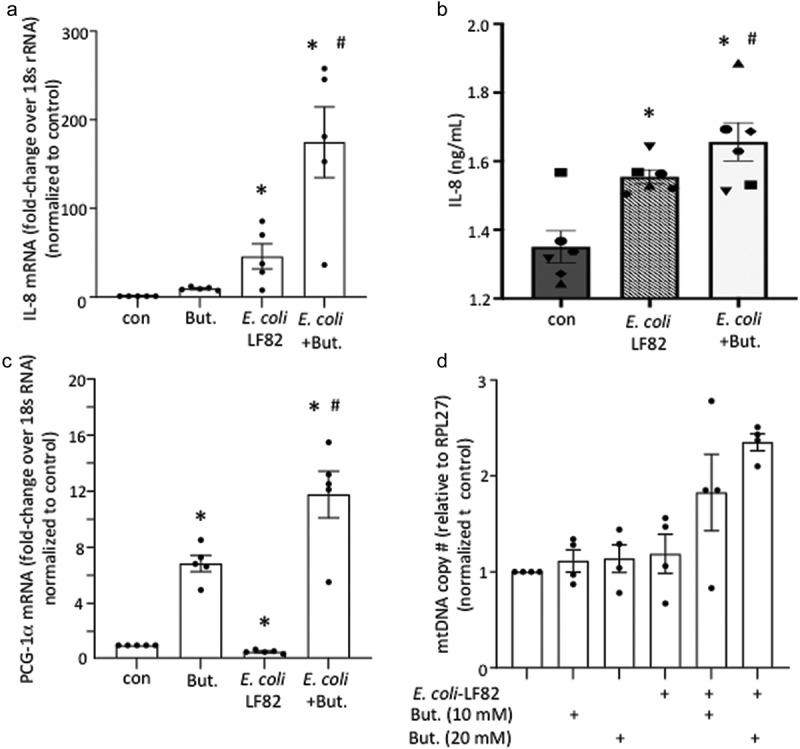
Butyrate and *E. coli*-LF82 increase epithelial transcription of IL-8 and PGC-1α. T84 epithelial cell monolayers exposed to *E. coli*-LF82 (10^8^ cfu, 4 h) showed increased IL-8 mRNA and protein that was enhanced by co-treatment with butyrate (But., 10 mM) (a, b). Expression of the master regulator of mitochondrial synthesis, peroxisome proliferator-activated receptor gamma co-activator 1-alpha (PGC-1α), was significantly increased by butyrate, and more so in the presence of *E. coli*-LF82, with a trend toward increased mitochondrial DNA (mtDNA) copy number (c, d) (data are mean ± SEM; *n* = 5–4; * and #, *p* <0.05 compared to control (con) uninfected cells and *E. coli*-LF82 only infected cells respectively by two-way ANOVA followed by Tukey’s multiple test; mRNA target genes were compared to expression of 18s rRNA as a housekeeping gene and the data normalized against control conditions set at 1).

Confirming published studies,^40^ *E. coli*-LF82-infected T84 cells showed a significant increase in IL-8 mRNA that was increased further by butyrate co-treatment (Figure 3a). This transcriptional effect was supported by a small statistically significant increase in IL-8 protein in *E. coli*-LF82+butyrate treated epithelia compared to *E. coli*-LF82 treatment only (Figure 3b). Increased IL-8 mRNA has been shown for *E. coli*-LF82 infected epithelia,^40^ and can also be a result of reduced mitochondrial activity and AMPK signaling.^22,41^ The reduction in the expression of the master regulator of mitochondrial synthesis, PGC-1α, caused by *E. coli*-LF82 observed in an RNA-sequence analysis^19^ was confirmed by qPCR (Figure 3c). Butyrate up-regulated expression of PGC-1α in T84 epithelia and this was further enhanced in *E. coli*-LF82+butyrate co-treated T84 cells (Figure 3c). This was paralleled by a trend toward increased mitochondrial-DNA copy number (Figure 3d).

### E. coli*-LF82+butyrate-treated epithelia have mitochondrial membrane potentials and ATP levels similar to uninfected cells*

**Figure 4.**
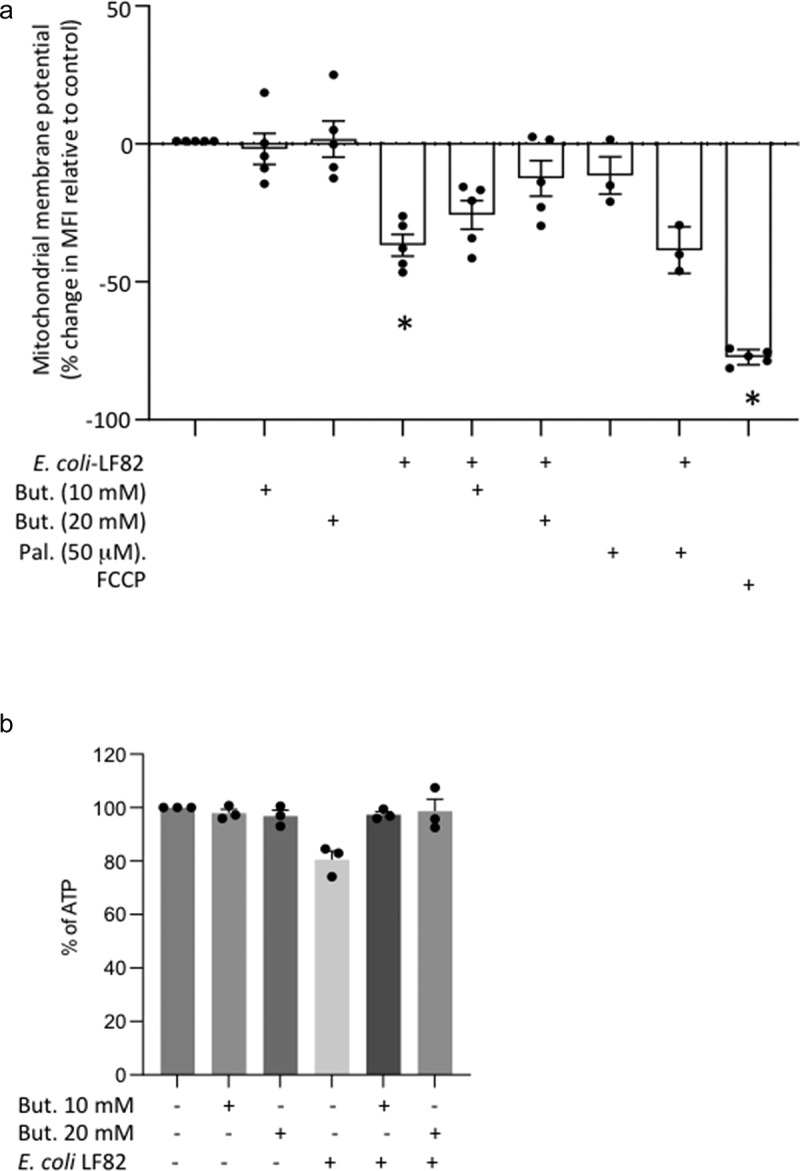
Butyrate co-treatment prevents *E. coli*-LF82 evoked loss of mitochondrial membrane potential and reduced intracellular ATP. (a) T84 epithelial cells were treated with *E. coli*-LF82 (10^8^, cfu) ± butyrate (But.) or palmitate (Pal.) for 4 h and then membrane potential was assessed by TMRE fluorescence in a flow cytometer. A 10 min treatment with the metabolic toxin, FCCP (10 μM) was used as a positive control (*n* = 5, except Pal. where *n* = 3)). (b) ATP was measured in epithelial lysates by commercial assay in triplicate in three experiments (data are mean ± SEM; *, *p* < .05 compared to control uninfected cells by the Kruskal–Wallis test followed by Dunn’s multiple comparison test for normalized data; statistics not performed on data in panel B because *n* = 3; MFI, mean fluorescent intensity).

Loss of mitochondrial membrane potential (ΔΨ_M_) is a pro-fission stimulus. In this acute infection paradigm (10^8^ cfu, 4 h), *E. coli*-LF82-infected epithelia displayed a drop of 30–40% ΔΨ_M_ compared to controls, that was either not apparent or significantly less in butyrate co-treated cells (Figure 4a). *E. coli*-LF82+palmitate treated T84 cells showed a significant drop in ΔΨ_M_ (Figure 4a), in accordance with the inability of this LCFA to prevent the bacteria-evoked mitochondrial fragmentation (Suppl. Fig. S4). The reduced intracellular ATP levels observed in *E. coli*-LF82-infected T84 cells were not observed in cells co-treated with butyrate (Figure 4b).

### *Butyrate modulates metabolism of epithelial-*E. coli *co-cultures*

**Figure 5.**
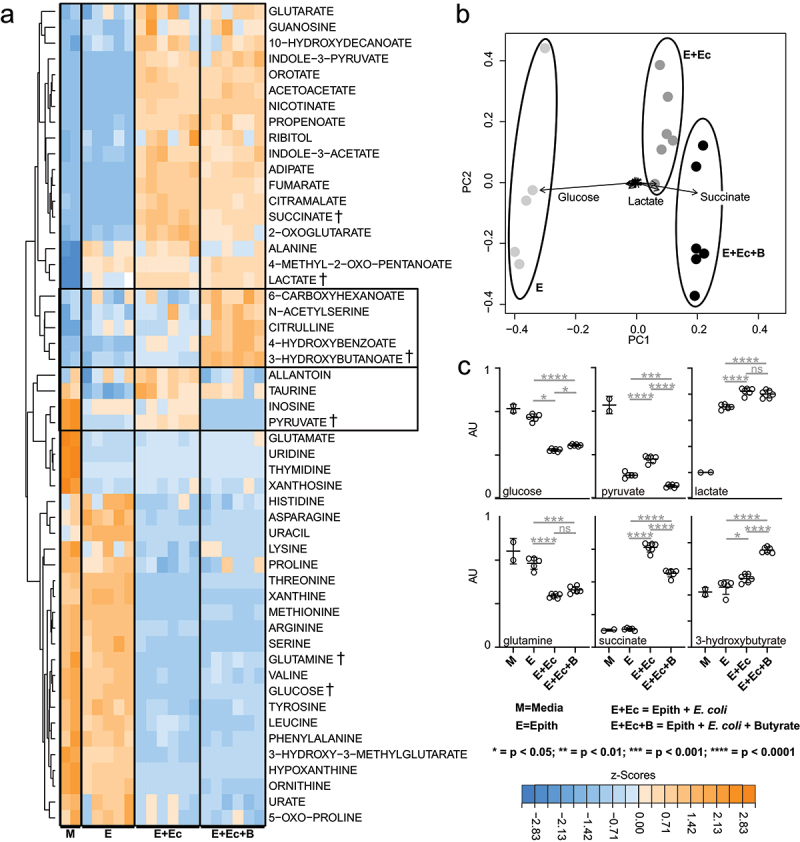
Butyrate modulates metabolism in *E. coli*-LF82 infected T84 epithelial cells. The metabolic activity of *E. coli*-LF82 infected (10^8^ cfu, 4 h) and non-infected T84 epithelial (epith) cells ± butyrate (10 mM) was assessed by LC-MS analyses of culture media. Metabolites that were consumed or produced by cultures were identified and quantified. (a) heat-map of 52 media metabolites with signal intensities shown as z-scores (*i.e.*, mean centered, variance stabilized signal intensities). Boxed metabolites indicate clusters of butyrate-linked metabolic perturbations. A selection of representative metabolites (†) illustrating the main metabolic patterns are shown as dot-plots in panel 5C. (b) Principal component analysis (PCA) of metabolite signals with the magnitude and direction of metabolite contributions shown as vectors (in a bi-plot). The three largest metabolic contributors to clustering are annotated above the vectors. (c) Representative metabolite levels (data show as mean ± SEM for *n* = 6; AU, arbitrary units) are plotted with significant differences denoted as calculated by pairwise t-test.

Adherent T84 cell cultures infected with *E. coli* were incubated for 4 h ± butyrate and metabolic fluxes were analyzed by LC-MS.^30,32^ Analysis showed that the metabolic phenotypes of non-infected T84 cells are distinct from those co-cultured with *E. coli*-LF82 (Figure 5a, b). The patterns of consumed versus secreted metabolites in the *E. coli-*LF82+T84 cell cultures closely followed the microbial phenotypes (Suppl. Fig. S5), indicating that bacterial metabolism was the primary driver in the co-cultures. However, lactate levels (a metabolite produced at low levels by *E. coli* under aerobic conditions but significantly produced by mammalian cells) matched levels seen in the T84 cells (Figure 5c), suggesting increased epithelial glycolytic metabolism. The effect of butyrate on epithelial-bacterial co-cultures largely mirrored the phenotypes seen in *E. coli*-LF82 only cultures. The addition of butyrate diminished glucose utilization, diminished succinate secretion, and stimulated 3-hydroxybutyrate production. As expected, lactate levels did not respond to butyrate in the co-culture conditions (Figure 5c). Thus, metabolomics experiments showed that microbial metabolism has a major impact on overall fluxes in the *E. coli*-LF82/T84 cell co-culture system, and that butyrate exposure affects the abundance of microbe-derived succinate and other metabolites that are linked to mitochondrial metabolism.

### *Butyrate co-treatment reduces* E. coli*-LF82 epithelial translocation*

**Figure 6.**
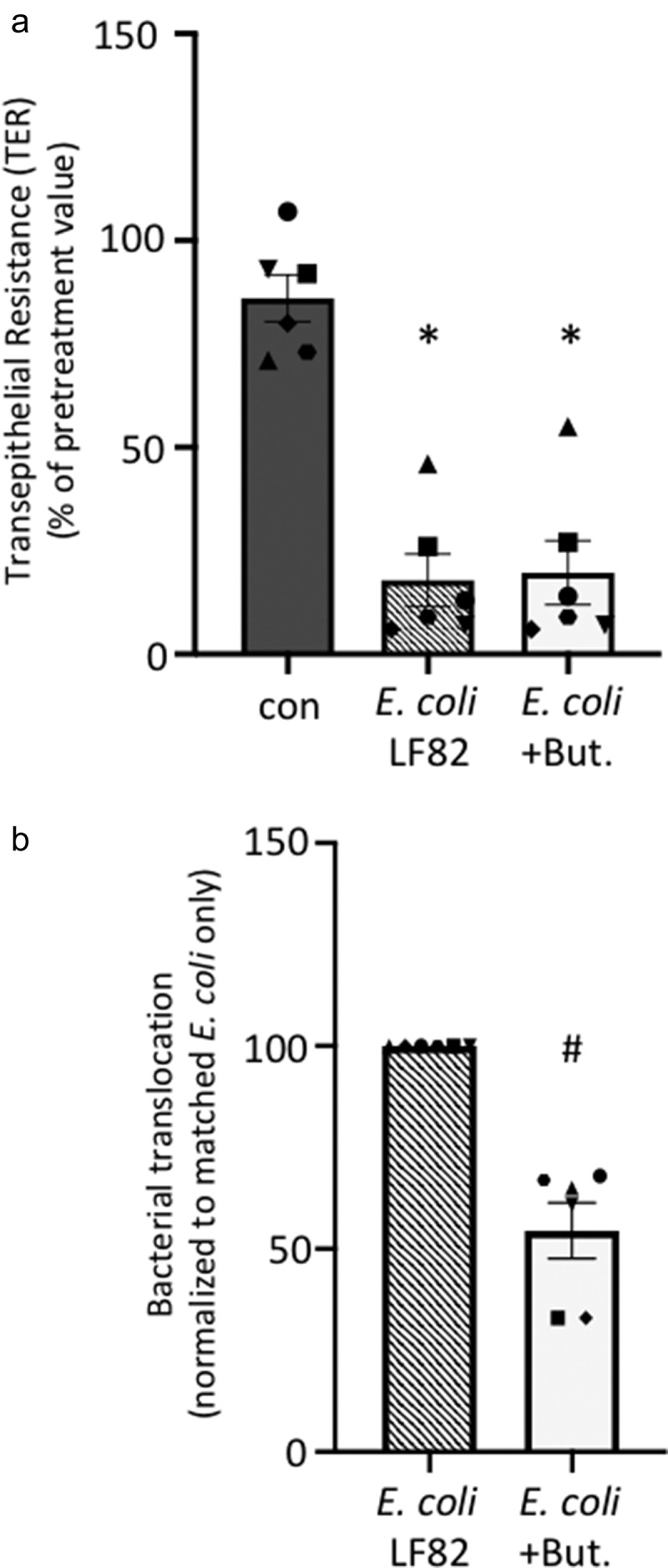
*E. coli*-LF82 translocation across epithelia is less pronounced in butyrate co-treated epithelia. Human colon-derived T84 cells (10^6^) were seeded onto porous (3 μm) transwell supports and cultured until electrically confluent, typically 7 days when transepithelial resistance exceeded 750 Ohms.cm^2^. *E. coli*-LF82 (10^8^ cfu) ± butyrate (But., 10 mM) were added to the apical surface and TER and transcytosis of the bacteria assessed 24 h later. (a) TER is presented as the change after 24 h with each monolayer being its’ own control (*i.e*., pretreatment value). Starting TERs in these experiments ranged from 750–2460 Ohms.cm^2^. (b) Bacterial transcytosis was assessed via serial dilution of culture-well basolateral medium on agar plates, with the data being converted to % transcytosis based on bacterial counts in the apical compartment. *E. coli*-LF82 data were normalized to 100 for comparison with *E. coli*+But. in the same experiment (data are mean ± SEM; each data point is an individual experiment (*n* = 6) in which bacterial transcytosis across 3 or 4 epithelial monolayers was averaged and are represented by a different symbol; * and #, *p* <.05 compared to control uninfected cells (con) and *E. coli*-LF82 only infected cells, respectively).

Transepithelial resistance (TER) reflects the ionic permeability of the tight junction and is used as a marker of the paracellular passage of small molecules. In six experiments, TER measurements at 4 h revealed no consistent significant differences between the groups, whereas by 24 h post-infection with *E. coli*-LF82 resulted in a reduction of TER by 54–96% of pre-treatment levels (Figure 6a). Butyrate co-treatment did not prevent the *E. coli*-LF82 evoked drop in TER across T84-cell monolayers (Figure 6a). Examining the passage of *E. coli*-LF82 across the epithelial layer after 4 h revealed a small, and perhaps paradoxical increase in *E. coli*+butyrate treated monolayers (data not shown); however, by 24 h post-treatment, butyrate co-treatment had reduced bacterial translocation by ~ 45%. Given the lack of a protective effect of butyrate co-treatment on TER we speculate that the bacteria translocation was likely via a transcellular route.

### Butyrate rescues mitochondrial network morphology via FFAR3

Butyrate acts in three major ways: as an energy source; an inhibitor of histone deactylase activity (HDAC); or by GPCR signaling.^4^ Use of an etomoxir co-treatment to block fatty acid oxidation, and hence butyrate’s role as an energy source, failed to prevent butyrate from preserving epithelial mitochondrial network structure (*n* = 5) or ΔΨ_M_ in the face of challenge with *E. coli*-LF82 (Suppl. Figs. S6 & 7; etomoxir bioactivity confirmed in the Agilent Seahorse Cell Metabolic Analysis – XF Analyzer).

Assessing a role for HDAC inhibition, trichostatin-A (TSA) was tested as a well-characterized HDAC inhibitor that targets the range of HDACs sensitive to butyrate.^42^ Trichostatin-A did not affect *E. coli* growth or invasion of T84 epithelia (data not shown, 4 epithelia from 2 experiments), and maintained a more fused mitochondrial network, with a lower mitochondrial fragmentation count and a corresponding increase in average mitochondrial length in cells co-treated with *E. coli*-LF82 (Suppl. Fig. S6). This effect was reminiscent of the effect of butyrate; however, unlike butyrate, TSA did not preserve the Ψ_M_ (Suppl. Fig. S7). Thus, while HDAC inhibition cannot be ruled out as contributing to butyrate’s effect on mitochondrial dynamics, it is unlikely to be the sole mechanism underlying butyrate’s antagonism of *E. coli*-LF82-evoked fragmentation of the epithelial mitochondrial network.

**Figure 7.**
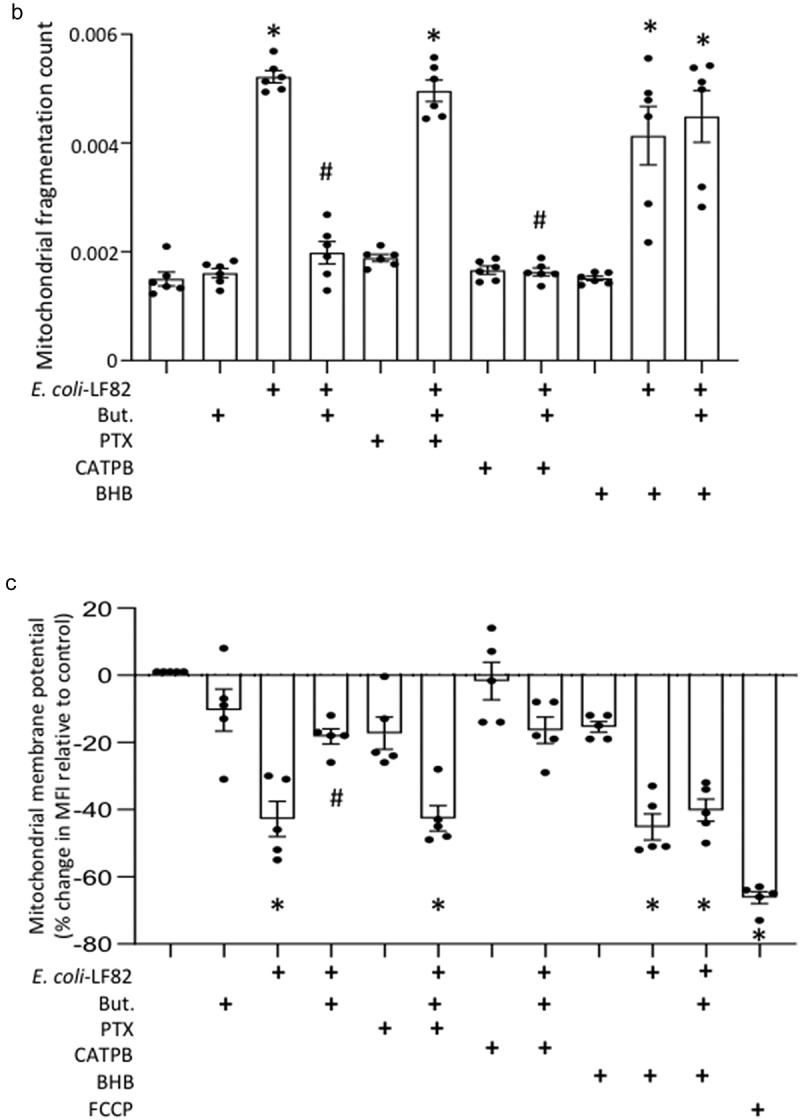
(continue).

Butyrate is a ligand for FFAR2 (or GPR-43), FFAR3 (or GPR-41) and HCAR (or GPR-109A). FFAR3 and HCAR are Gi/o-family proteins and are sensitive to pertussis toxin (PTX), while FFAR2, is a Gq/Gi family member: mRNA for all three receptors was demonstrated in T84 epithelia (Suppl. Fig. S 8A,B). Pertussis toxin, but not the FFAR2 selective antagonist CATPB, blocked the ability of butyrate to limit *E. coli*-LF82-evoked mitochondrial fragmentation, mitochondrial fragmentation count and ΔΨ_M_ (Figure 7a–c). These data implicate either FFAR3 or HCAR in mediating the effects of butyrate.
**Figure 7.**
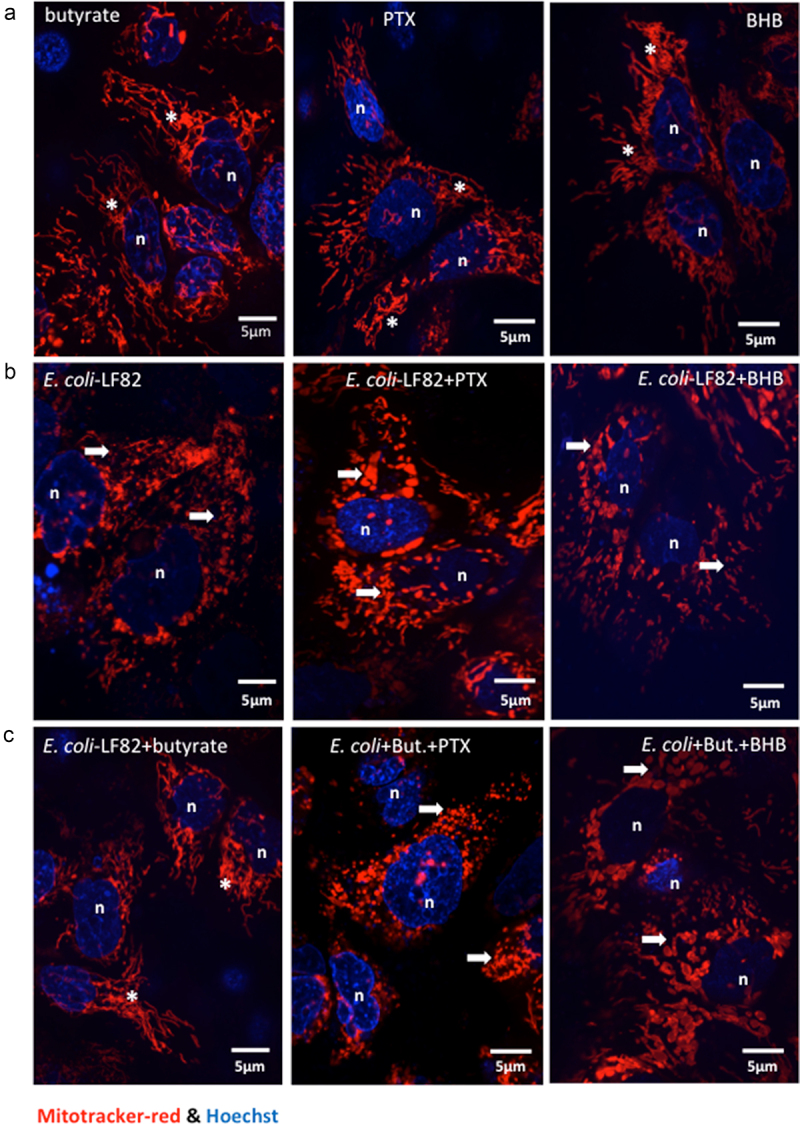
Pertussis toxin and β-hydroxybutyrate (BHB) implicate FFAR3 in butyrate’s maintenance of epithelial mitochondrial networks. Monolayers of the human colon-derived T84 epithelial cell line (10^6^) were treated with *E. coli*-LF82 (10^8^ cfu, 4 h) ± a co-treatment with sodium butyrate (But., 10 mM) ± an 18 h pre-treatment with pertussis toxin (PTX, 50 ng/ml) or BHB (5 mM). (a) Representative images were collected in a random fashion by first identifying epithelia nuclei (blue, n) and then swapping the confocal laser channel to assess the mitochondrial network (Mitotracker (red)). Twenty cells per monolayer were characterized for mitochondrial fragmentation count by ImageJ analysis and averaged/monolayer (b). (c) Mitochondrial membrane potential was assessed by TMRE fluorescence in a flow cytometer. A 10 min treatment with the metabolic toxin, FCCP (10 Ohms.cm^2^) was used as a positive control. The effect of the FFAR2 antagonist (S)-3-(2-(3-chlorophenyl) acetamido)-4-(4-(trifluoromethyl) phenyl) butanoic acid (CATPB, 10 Ohms.cm^2^, 30 min pre-treatment) is also shown (mean ± SEM; *n* = 5–6 epithelial monolayers from separate experiments in panels a and b; * and #, *p* <.05 compared to control uninfected cells and *E. coli*-LF82 only infected cells respectively by two-way ANOVA followed by Tukey’s multiple comparison test (b) or the Kruskal–Wallis test from by Dunn’s multiple comparison test; image *, fused mitochondrial network with elongated strands; arrow, fragmented, vesiculated area of the mitochondrial network; MFI, mean fluorescence intensity).

To distinguish between FFAR3 and HCAR, we tested the effect of β-hydroxybutyrate (BHB), an antagonist of FFAR3 and contrarily, an agonist of HCAR. Substantial mitochondrial fragmentation and loss of Ψ_M_ occurred in *E. coli*-LF82+BHB treated T84 cells suggesting that BHB activation of HCAR did not rescue the mitochondrial dysfunction (Figure 7). In contrast, when BHB was applied to *E. coli*-LF82+butyrate treated T84 cells, the protective effect of butyrate was lost, and mitochondrial fragmentation was obvious. These data support the postulate that BHB antagonism of FFAR3 ablates the ability of butyrate to reduce the effect of *E. coli*-LF82 on T84-cell mitochondrial network morphology (Figure 7).

**Figure 8.**
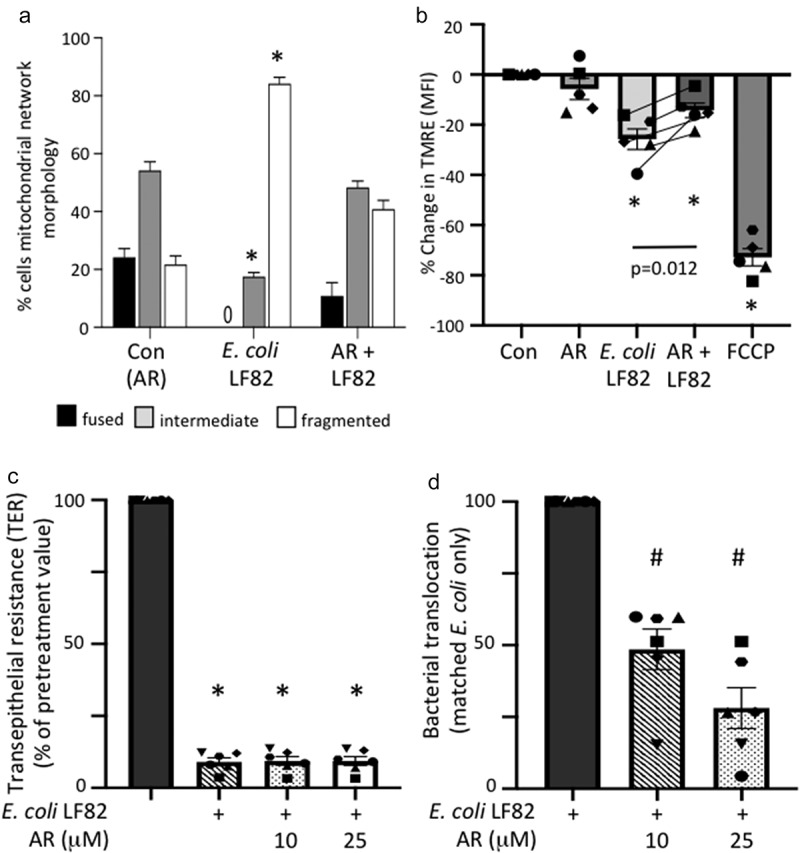
An FFAR3 agonist improves mitochondrial network connectivity and barrier function in *E. coli*-LF82-infected T84 epithelial cells. Monolayers of the human colon-derived T84 epithelial cell line were treated with *E. coli*-LF82 (10^8^ cfu, 4 h) ± a co-treatment with the FFAR3 agonist AR420626 (25 µM) and representative images collected in a random fashion by first identifying epithelia nuclei (blue, n) and then swapping the confocal laser channel to assess the mitochondrial network as defined by TOMM-20 immunostains. Twenty cells per monolayer were characterized by semi-quantitative assessment (a). (b) Mitochondrial membrane potential was assessed by TMRE fluorescence in a flow cytometer. A 10 min treatment with the metabolic toxin, FCCP (10 µM, *n* = 5 epithelial monolayers from separate experiments (indicated by different symbols)). Filter-grown T84 cell monolayers (starting transepithelial resistance (TER) range = 957–2155 Ohms.cm^2^) were cultured with *E. coli*-LF82 (10^8^ cfu) ± AR420626 and TER and transcytosis of the bacteria assessed 24 h later. (c) TER is presented as the change over 24 h with each monolayer being its’ own control (*i.e*., pre-treatment value). (d) Bacterial transcytosis was assessed via serial dilution of culture-well basolateral medium on agar plates, with the data being converted to % transcytosis based on bacterial counts in the apical compartment and then *E. coli*-LF82 was normalized to 100 for comparison with *E. coli*+AR420626 in the same experiment (data are mean ± SEM; each data point is an individual experiment (*n* = 6) in which measurements from 3 or 4 monolayers were averaged and are shown as a different symbol; * and #, *p* <.05 compared to control uninfected cells (con) and *E. coli*-LF82 only infected cells, respectively).

**Figure 9.**
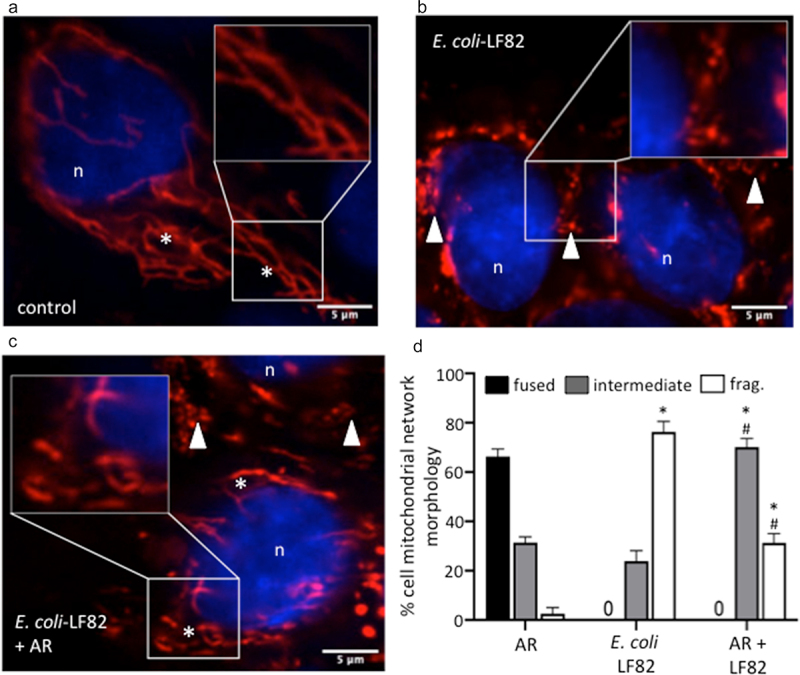
An FFAR3 agonist improves mitochondrial network connectivity in *E. coli*-LF82-infected human organoids. Monolayers of colonic organoids derived from two healthy controls were treated with *E. coli*-LF82 (MOI = 100, 4 h) ± a co-treatment with the FFAR3 agonist AR420626 (AR: 25 µM). Representative confocal images of MitoTracker^TM^ (red) and Hoescht (blue) co-stained organoids show the fused mitochondrial network of control organoid cells treated with the FFAR3 agonist alone (a), the puncta-like fragmented mitochondrial network of *E. coli*-LF82 infected organoid cells (b), and the intermediate fragments of the *E. coli-*LF82 infected organoids co-treated with the FFAR3 agonist (c) (n, nucleus; *, filamentous mitochondria; arrowhead, fragmented mitochondria). Twenty cells were assessed from four monolayers for semi-quantitative analysis (d). Data are mean ± SEM, * and #, *p* < .05 compared to control uninfected drug-treated cells (AR) and *E. coli*-LF82 only infected cells, respectively (two-way ANOVA followed by Tukey’s multiple comparison test) (frag., fragmented mitochondria).

FFAR3 may be expressed on a range of epithelial sub-types. Immunoblotting confirmed the expression of FFAR3-protein on human organoids and the T84, CaCo2 and HT-29 human colon-derived epithelial cell lines (Suppl. Fig. S8C). Subsequently, use of the FFAR3-selective agonist, AR420626, revealed substantial preservation of the mitochondrial network in *E. coli*-LF82 co-treated cells (Figure 8a) and partial maintenance of Ψ_M_ (Figure 8b). Similar to butyrate, AR420626 did not affect the *E. coli*-LF82-evoked drop in TER, but significantly reduced the transcytosis of the bacteria across filter-grown T84 epithelial monolayers when assessed 24 h post-infection (Figure 8d). Finally, the effect of AR420626 was tested in organoid+*E. coli*-LF82 co-cultures (Figure 9). As previously, *E. coli*-LF82 evoked dramatic mitochondrial fragmentation in the organoid epithelium and in this set of experiments no cells with a fused network were observed. Co-treatment with the FFAR3 agonist resulted in significant preservation of the epithelial mitochondrial network. While long elongated fused mitochondrial networks were not apparent in *E. coli*-LF82+AR420626 treated organoids there was a significant increase in the number of cells with an intermediate phenotype and fewer with massively fragmented mitochondrial networks (Figure 9).

## Discussion

The epithelial lining of the gut serves as a barrier, a sentinel and a regulator of mucosal immunity. These functions are energy dependent,^43,44^ and we and others have identified perturbed mitochondrial form, function and transcriptomics in tissue from individuals with IBD.^12–14,36^ Many pathogens, including the Crohn’s disease-associated pathobiont, *E. coli*-LF82 significantly perturb epithelial mitochondrial function.^18,45^ The anti-inflammatory and barrier-enhancing effects of butyrate demonstrated by use of butyrate-producing bacteria and butyrate in murine models of colitis and *in vitro*^23,46,47^ raised the possibility that this SCFA may antagonize the *E. coli*-LF82-evoked epithelial mitochondrial fragmentation. This may be particularly pertinent in the context of IBD, which is often accompanied by dysbiosis characterized by loss of SCFA-producing species of bacteria.^5,20^ The data herein support this position, revealing preservation of mitochondrial form/function in the face of infection with an AIEC as another benefit of butyrate, achieved, at least in part, through FFAR3.

Butyrate, at levels reported in the colon, significantly reduced the mitochondrial fragmentation caused by the AIEC, *E. coli*-LF82 in T84 and CaCo2 epithelial cell lines (confirming earlier studies)^19^and in human primary epithelial colonoids. While the majority of cells in the organoids are transporting enterocytes we should not overlook the possibility that specialty epithelia within the organoid (e.g. goblet cells) could be more or less sensitive to the presence of *E. coli*-LF82 or butyrate: a topic deserving of future research efforts. In addition, since *E. coli*-LF82 can survive within macrophages^8^ and macrophages respond to butyrate,^3,48,49^ there is value in assessing mitochondrial form and function in infected macrophages and any impact butyrate may have on this aspect of pathobiont–macrophage interaction.

Butyrate-evoked killing of the *E. coli* would diminish the stimulus to elicit mitochondrial fragmentation because viable bacteria are essential to observe this impact on the enterocyte. In this context, at pH 6.5, butyrate decreased *E. coli*-LF82 growth and at pH 7.2 decreased invasion into CaCo2 cells.^50^ This was not the case for T84-epithelia were the SCFA was neither bacteriostatic nor bactericidal, and did not reduce bacterial invasion of T84-epithelia. However, metabolomic analysis reveals that the *E. coli* were not oblivious to the presence of butyrate and so altered gene expression in the bacteria cannot, at this stage, be unequivocally ruled out as a possible contributing factor to the preservation of epithelial mitochondrial network morphology.

Mechanistically, butyrate diminished the loss of mitochondrial membrane potential (Ψ_M_) evoked by *E.* *coli*-LF82, removing a mitochondrial pro-fission signal. Loss of Ψ_M_ has been noted with other microbial pathogens,^51,52^ including uropathogenic *E. coli*,^53^ suggesting that butyrate’s maintenance of Ψ_M_ could be protective against a variety of bacteria. The metabolomics data show that butyrate exposure directly affects levels of succinate secreted by *E. coli*-LF82. Elevated succinate concentrations (and other tricarboxylic acid cycle (TCA)-linked metabolites) in mammalian cells may perturb mitochondrial transport fluxes (e.g. malate/aspartate shuttle) and thereby lead to loss of Ψ_M_.^54^ The ameliorating effect of butyrate on microbial succinate secretion may help bring the mammalian intracellular equilibrium back sufficiently to limit mitochondrial fragmentation: an hypothesis requiring testing.

In addition to limiting a pro-fission stimulus, the increased expression of PCG-1α in butyrate-treated T84 cells would promote biogenesis of mitochondria. In this context, pigs fed butyrate had increased PGC-1α in muscle tissue,^55^ and butyrate sustained PCG-1α expression in the porcine intestinal epithelial IPEC-J2 cell line challenged with H_2_O_2_, while promoting mitophagy via an AMPK-dependent mechanism to clear damaged mitochondrial fragments.^56^ Generation of new mitochondria would be expected to increase overall cellular Ψ_M_, leading to improved energetics and function.

Although butyrate can be catabolized into acetyl-CoA and thereby supply carbon to the TCA cycle, the acyl-CoA transport inhibitor, etomoxir, did not affect butyrate’s maintenance of the mitochondrial network or ∆Ψ_M_ in the presence of *E. coli*-LF82. These data suggest that the protective effect of butyrate was not due to the direct catabolism of butyrate and its role as an energy source.

Butyrate regulates gene transcription by inhibiting HDACs to promote DNA hyper-acetylation.^57^ Since butyrate increased T84-cell expression of IL-8 and PGC-1α mRNA, the possibility that it ameliorated *E. coli*-LF82 induced mitochondrial fragmentation via HDAC inhibition was considered. Co-treatment of *E. coli*-LF82-infected T84 cells with the HDAC inhibitor, trichostatin-A (TSA), resulted in significant improvement in mitochondrial network architecture. Butyrate and TSA target the same HDACs suggesting that butyrate’s action as a HDAC inhibitor could underlie its effect on the mitochondria; however, while limiting *E. coli*-LF82 evoked disruption of the mitochondrial network, TSA-treated cells still displayed reduced ∆Ψ_M_. This is a curious finding deserving of future research, but it suggests that butyrate’s effect on the enterocyte’s mitochondria is not solely via HDAC inhibition, but may underlie the increased expression of PGC-1α mRNA.^58^ Since neither a role as an energy source nor as a HDCA inhibitor provided a satisfactory explanation for butyrate’s maintenance of epithelial mitochondrial form and function in the current experimental paradigm, its role as a GPCR ligand was examined.

Enteric epithelial cells express GPCRs for SCFAs with some promiscuity in binding acetate, propionate and butyrate.^59^ Ablation of butyrate’s mitochondrial protective effect by pertussis toxin pointed to GPCR involvement. In contrast, PTX did not affect the up-regulation of IL-10 Rα mRNA in butyrate-treated T84 cells,^42^ underscoring the complexity of the epithelial-SCFA relationship. Experiments with the FFAR3 antagonist, β-hydroxybutyrate in T84 epithelia, and the FFAR3 agonist, AR420626, in T84 cells and human organoids, blocked and recapitulated the effect of butyrate, respectively. These findings support a hitherto unappreciated role for FFAR3 in preserving mitochondrial form/function and epithelial barrier function in bacterial pathobiont-infected epithelia. Little is known of FFAR3 biology in the gut^60^; thus, given the recognized benefits of SCFAs to host physiology,^61^ greater research on the FFARs/GPRs is warranted. For instance, the recent description of FFAR2/FFAR3 heterodimers^62^ is intriguing, and could add nuance to the understanding of the contribution of individual FFARs to a healthy gut. The possibility that FFAR3 agonists could lessen the impact of bacterial pathogens on enteric epithelial barrier function by maintaining mitochondrial form and function is a therapeutic possibility worthy of pursuit. In this instance, we speculate that maximal benefit of butyrate would occur via its HDAC inhibitory activity and signaling through GPCRs on the enterocyte.

Maintaining mitochondrial form and function when challenged with bacterial pathogens has wide-ranging implications for the cell, ranging from Ca^2+^ homeostasis, to control of ER-mitochondrial interaction and ER-stress, to ROS generation and intracellular signaling. Assessing IL-8 production, butyrate increased T84-cell IL-8 mRNA and protein, which *in vivo* would recruit neutrophils to combat bacterial infection. In addition, butyrate co-treated epithelia displayed reduced transepithelial passage of *E. coli*-LF82 indicating enhanced barrier function. There is precedent for SCFA maintenance of epithelial barrier function. For example, increased passage of commensal *E. coli* across T84 cells monolayers treated with the hydrogen ionophore, dinitrophenol (disrupts Ψ_M_) was reduced by butyrate and correlated with reduced NFκβ activation in that *in vitro* model.^23^ Also, up-regulation of PGC-1α in primary airway epithelial cells *in vitro* restored barrier function that had been reduced by rhinovirus.^63^

In summary, employing human organoids and established human colon-derived epithelial cell lines, the mitochondrial fragmentation caused by infection with the AIEC *E. coli*-LF82 was reduced in severity by co-treatment with butyrate, acting in part via FFAR3 (the possibilities of butyrate-evoked changes in metabolism or action as a HDAC inhibitor should not be dismissed). We speculate that dysbiosis characterized by loss of SCFA-producing species of bacteria or a reduced ability of enterocytes to respond to SCFA, via, for instance, diminished SCFA transporters^64^ or FFAR expression will leave the individual vulnerable to the emergence of pathobionts with the capacity to disrupt epithelial mitochondrial and gut barrier functions.

## Supplementary Material

Supplemental Material
