# Supplementary material for: Butyrate reduces adherent-invasive E. coli-evoked disruption of epithelial mitochondrial morphology and barrier function: involvement of free fatty acid receptor 3
Source: Gut Microbes. 2023 Dec 11;15(2):2281011. doi: 10.1080/19490976.2023.2281011 (PMC10730202; doi:10.1080/19490976.2023.2281011)
Supplement: Supplemental Material [file KGMI_A_2281011_SM8049.zip › KGMI_Supplemental figures and tables/Supplemental tables and figures legends.docx]

**Butyrate reduces adherent-invasive *E. coli*-evoked disruption of epithelial mitochondrial morphology and barrier function: involvement of free fatty acid receptor 3**

Samira A. Hamed, Armaan Mohan, Saranya Navaneetha Krishnan, Arthur Wang, Marija Drikic, Nicole L. Prince, Ian Lewis, Jane Shearer, Åsa V. Keita, Johan D. Söderholm, Timothy E. Shutt, and Derek M. McKay

**Supplementary Figure Legends**

**S. Figure 1. T84 epithelia display a normal mitochondrial network after exposure to commensal *E. coli*.** Confocal micrographs show the fused mitochondrial network in a representative T84 human colon-derived epithelial cell as visualized by Mitotracker^TM^ staining (red). A similar mitochondrial network architecture was consistently observed in cells exposed to the commensal *E. coli*, strain F18 (epithelial nuclei shown in blue).

**S. Figure 2: *E. coli*-LF82 evoked fragmentation of enteric epithelial mitochondria is reduced in butyrate co-treated cells.** Monolayers of the human colon-derived CaCo2 epithelial cell line (10^6^) were treated with *E. coli*-LF82 (10^8^ cfu, 4h) ± a co-treatment with sodium butyrate (But., 10 mM). Representative images (A) were collected in a random fashion by first identifying epithelia nuclei (blue, n) and then swapping the confocal laser channel to assess the mitochondrial network (Mitotracker (red)). Twenty cells per monolayer were characterized by semi-quantitative analysis (B) (data are mean ± SEM, from 4 monolayers assessed in 2 experiments; * and #, p<0.05 compared to control uninfected cells and *E. coli*-LF82 only infected cells, respectively by two-way ANOVA followed by Tukey’s multiple comparisons test; image *, fused mitochondrial network with elongated strands; arrow, fragmented, vesiculated area of the mitochondrial network).

**S. Figure 3: *E. coli*-LF82 evoked fragmentation of enteric epithelia is reduced by short chain but not long chain fatty acid co-treatment**. Monolayers of the human colon-derived T84 epithelial cell line were treated with *E. coli*-LF82 (10^8^ cfu, 4h) ± a co-treatment with acetate (Acet.; 3 mM), propionate (Prop.; 3 mM) (both short chain fatty acids) or the long-chain fatty-acid, palmitate (Palm.; 50 μM). Representative images were collected in a random fashion by first identifying epithelia nuclei (blue, n) and then swapping the confocal laser channel to assess the mitochondrial network (Mitotracker (red)). Twenty cells per monolayer were characterized by semi-quantitative analysis (B) (data are mean ± SEM, from 4-6 epithelial monolayers assessed in 2 experiments; * and #, p<0.05 compared to control uninfected cells and *E. coli*-LF82 only infected cells respectively by two-way ANOVA followed by Tukey’s multiple comparisons test; image *, fused mitochondrial network with elongated strands; arrow, fragmented, vesiculated area of the mitochondrial network; the effect of sodium butyrate co-treatment with *E. coli*-LF82 (But., 10 mM) in these experiments in shown in panel B).

**S. Figure 4: Altered mRNA expression for mitochondrial dynamics in biopsies from individuals with Crohn’s disease.** (A) Biopsies from controls (con: individuals under going colon cancer screening) or individuals with Crohn’s disease (inflamed, CD-i; non-inflamed, CD-ni) were obtained under ethical consent, and mRNA extracted and qPCR performed for specific target genes that were compared against the housekeeper gene ribosomal protein L27 (RPL27), and then normalized to control (data are mean ± SEM; *, p<0.05 compared to control (con) by one-way ANOVA followed by Dunnett’s test).

**S. Figure 5:** **Butyrate modulates metabolism in *E. coli*-LF82**. Analysis illustrating component consumption or metabolite production *E. coli*-LF82 (10^8^ cfu, 4h) ± butyrate (10 mM) cultures was assessed by LC-MS analyses of culture media. Metabolites that were consumed or produced by cultures were identified and quantified. (A) Heat-map of 52 media metabolites with signal intensities shown as z-scores (*i.e.,* mean centered, variance stabilized signal intensities). Boxed metabolites indicate clusters of butyrate-linked metabolic perturbations. A selection of representative metabolites (*) illustrating the main metabolic patterns are shown as dot-plots in panel C. (B) Principal component analysis (PCA) of metabolite signals with the magnitude and direction of metabolite contributions shown as vectors (in a bi-plot). The three largest metabolic contributors to clustering are annotated above the vectors. (C) Representative metabolite levels (data show as mean ± SEM for n=6; AU, arbitrary units) are plotted with significant differences denoted as calculated by pairwise t-test.

**S. Figure 6. A β-oxidation inhibitor does not prevent butyrate from maintaining a mitochondrial network in T84 epithelia co-treated with *E. coli*-LF82.** T84 epithelial cells (10^6^) were cultured with *E. coli*-LF82 (10^8^ cfu) ± butyrate (10 mM) for 4h ± a 30 min pre-treatment with etomoxir (ETO, 10 μM). Other monolayers were treated with *E. coli*-LF82 (10^8^ cfu) ± the HDAC inhibitor, trichostatin A (TSA, 2 μM, 30 min pre-treatment) for 4h. (A) Representative images were collected in a random fashion by first identifying epithelia nuclei (blue, n) and then swapping the confocal laser channel to assess the mitochondrial network (Mitotracker (red)). Twenty cells per epithelial monolayer were characterized by semi-quantitative analysis (B) and a mitochondria fragmentation count using an ImageJ analysis program (C) (data are mean ± SEM, from 6 epithelial monolayers from 3 experiments; * and #, p<0.05 compared to control uninfected cells and *E. coli*-LF82 only infected cells, respectively by two-way ANOVA followed by Tukey’s multiple comparisons test; image *, fused mitochondrial network with elongated strands; arrow, fragmented, vesiculated area of the mitochondrial network).

**S. Figure 7. Etomoxir and trichostatin-A do not protect epithelial mitochondria membrane potential in *E. coli*-LF82 infected cells.** T84 epithelial cells were treated with *E. coli*-LF82 (10^8^, cfu) ± butyrate (But.) ± a 30 min pre-treatment with the inhibitor of fatty acid oxidation, etomoxir (ETO, 10 μM) or the HDAC inhibitor, trichostatin A (TSA, 2 μM). Four hours later cells were prepared for flow cytometry and mitochondrial membrane potential assessed by TMRE fluorescence. A 10 min treatment with the metabolic toxin, FCCP (10 μM) was used as a positive control (data are mean ± SEM; n=4-5; * and #, p<0.05 compared to control uninfected cells and *E. coli*-LF82 only infected cells respectively by the Kruskal-Wallis test followed by Dunn’s multiple comparison test; MFI, mean fluorescence intensity).

**S. Figure 8. Human colonic epithelia express free fatty acid receptors (FFAR).** (A) Gel electrophoresis shows constitutive mRNA expression of FFAR2 and FFAR3 in T84 epithelia (n=4, 18s rRNA used as a loading control) and confirmed in (B) that shows CT values of real-time PCR for FFAR2, FFAR3 and HCAR. Panel C shows immunoblotting for FFAR3 in protein extracts from human duodenal (duoden.) and colonic organoids, and three commonly employed human colon-derived epithelial cell lines (protein loading: duoden. and colon organoids = 80 μg; T84 and CaCo2 cell lines = 40 μg; HT29 cell line,= 54 μg; mm, molecular marker; KDa, kilodalton).

**Supplementary Table 1:** healthy volunteer and patient with Crohn’s disease basic demographics

| ***Condition*** | ***Sex*** | ***Age*** | ***Disease Location*** | ***Disease State*** | ***Biopsy Location*** |
| --- | --- | --- | --- | --- | --- |
| Healthy | M | 50 | na | control | ascending colon |
| Healthy | M | 51 | na | control | descending colon |
| Healthy | F | 58 | na | control | descending colon |
| Healthy | F | 52 | na | control | ascending colon |
| Healthy | M | 59 | na | control | ascending colon |
| Healthy | F | 53 | na | control | ascending colon |
| Healthy | M | 65 | na | control | ascending colon |
| Healthy | M | 72 | na | control | ascending colon |
| Healthy | F | 51 | na | control | ascending colon |
| Healthy | M | 70 | na | control | ascending colon |
| Healthy | F | 44 | na | control | descending colon |
| Healthy | F | 28 | na | control | descending colon |
| Healthy | F | 19 | na | control | descending colon |
| Healthy | F | 63 | na | control | ascending colon |
| Healthy | F | 43 | na | control | ascending colon |
| CD | F | 47 | ileocolonic | non-inflamed | not noted |
| CD | F | 47 | ileocolonic | inflamed | not noted |
| CD | F | 25 | ileocolonic | non-inflamed | not noted |
| CD | F | 62 | ileocolonic | non-inflamed | not noted |
| CD | F | 39 | ileocolonic | inflamed | descending colon |
| CD | F | 39 | ileocolonic | non-inflamed | descending colon |
| CD | M | 64 | colonic | non-inflamed | transverse colon |
| CD | F | 31 | ileal | inflamed | neo-terminal ileum |
| CD | M | 50 | colonic | inflamed | sigmoid colon |
| CD | M | 50 | Colonic | non-inflamed | sigmoid colon |
| CD | M | 28 | ileal | inflamed | neo-terminal ileum |
| CD | F | 33 | ileocolonic | inflamed | descending colon |
| CD | F | 61 | unknown | non-inflamed | not noted |
| CD | M | 46 | ileocolonic | inflamed | not noted |
| CD | F | 50 | ileocolonic | non-inflamed | descending colon |

**Supplementary Table 2**: QPCR primer sequences (human) used in this study

| ***Gene*** | ***Primer Sequence*** |
| --- | --- |
| 18s RNA | forward: ACGCGCGCTACACTGACTGG  reverse: CGATCCGAGGGCCTCACTAAACC |
| free fatty acid receptor (FFAR)-2 (GPR-43) | forward: AAGGAGAAGGGATGCCAAGT  reverse: GGGATACCAAGCTGGTGAAA |
| FFAR-3 (GPR-41) | forward: GCTCAACTTCCTTGTGCTT  reverse: ACCCGGAGGAGGAGAAGTAGTA |
| hydroxcarboxylic acid receptor (HCAR) (GPR-109A) | forward: TGTTGGGGCTGGAGTTTATC  reverse: GTCCCAACGCCTCACATAGT |
| interleukin-8 | forward: GCAGCTCTGTGTGAAGGTGC  reverse: AAAGGTTTGGAGTATGTCTTTATGCA |
| mitochondrial 16s rRNA | forward: GCCTTCCCCCGTAAATGATA  reverse: TTATGCGATTACCGGGCTCT |
| peroxisome proliferating activating receptor gamma co-activator (PGC)-1α | forward: CAGAGGCAGAAGGCAATTGAAG  reverse: CTGTCCGTGTTGTGTCAGGT |
| ribosomal protein L27 (RPL27) | forward: ATCGCCAAGAGATCAAAGATAA  reverse: TCTGAAGACATCCTTATTGACG |
| dynamin-related protein-1 (Drp1) | forward: ACCCGGAGACCTCTCATTCT  reverse: TGACAACGTTGGGTGAAAAA |
| mitochondrial fission-1 (Fis1) | forward: GACATCCGTAAAGGCATCCGT  reverse: GAAGACGTAATCCCGCTGTT |
| mitofusion-1 (MFN1) | forward: GTGAGCTTCACCAGTGCAAAA  reverse: CATTGTGCTGAATAACCCTCAA |
| optic atrophy factor-1 (Opa1) | forward: TCCCGCTTTATGACAGAACC  reverse: AAATAGCTGCATCCCATTGC |
