# Supplementary figures and images for: Butyrate reduces adherent-invasive E. coli-evoked disruption of epithelial mitochondrial morphology and barrier function: involvement of free fatty acid receptor 3
Source: Gut Microbes. 2023 Dec 11;15(2):2281011. doi: 10.1080/19490976.2023.2281011 (PMC10730202; doi:10.1080/19490976.2023.2281011)

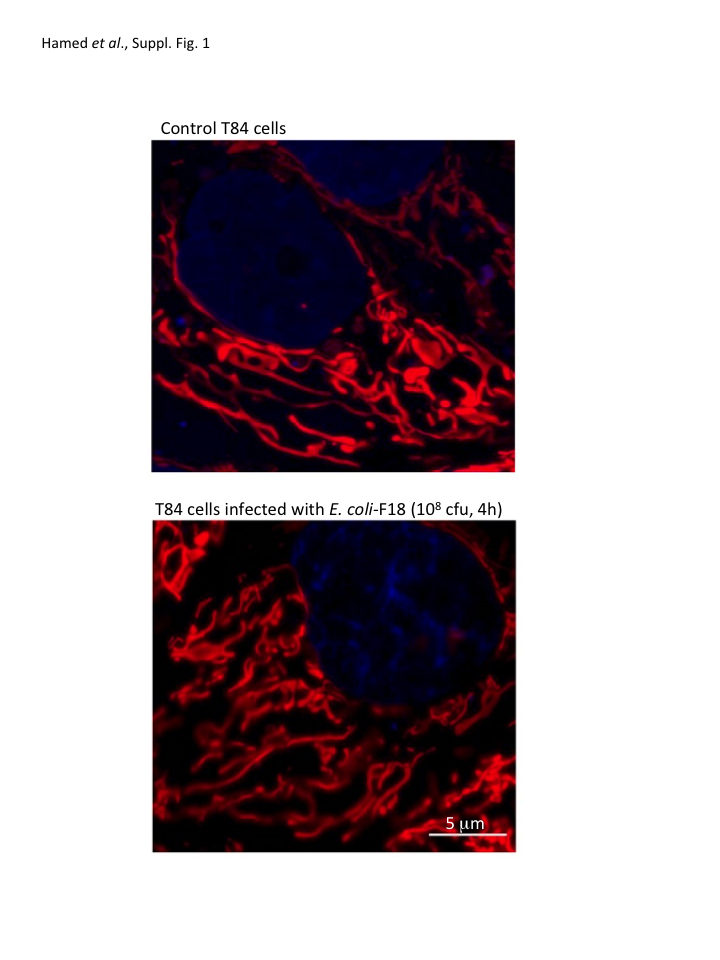

Supplement: Supplemental Material [file KGMI_A_2281011_SM8049.zip › KGMI_Supplemental figures and tables/Hamed Fig S1.tiff]

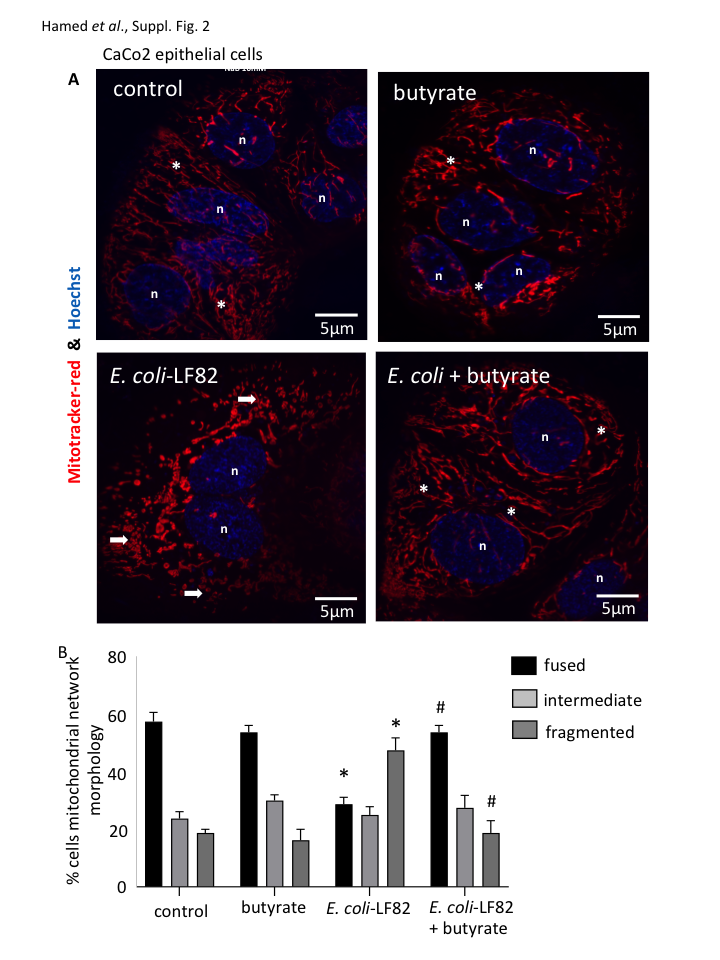

Supplement: Supplemental Material [file KGMI_A_2281011_SM8049.zip › KGMI_Supplemental figures and tables/Hamed Fig S2.tiff]

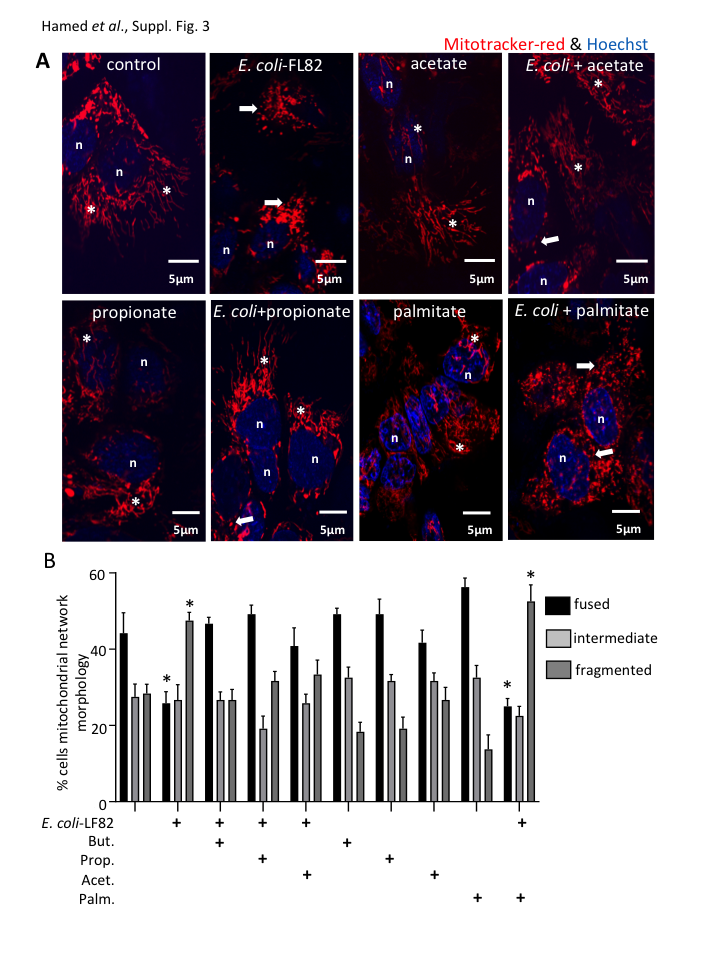

Supplement: Supplemental Material [file KGMI_A_2281011_SM8049.zip › KGMI_Supplemental figures and tables/Hamed Fig S3.tiff]

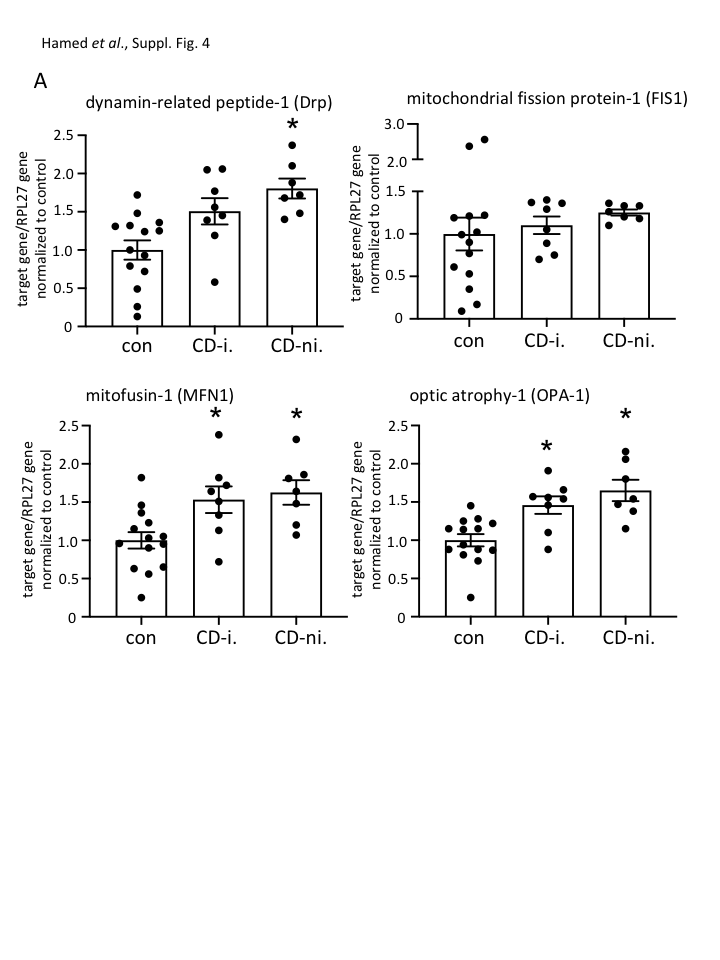

Supplement: Supplemental Material [file KGMI_A_2281011_SM8049.zip › KGMI_Supplemental figures and tables/Hamed Fig S4.tiff]

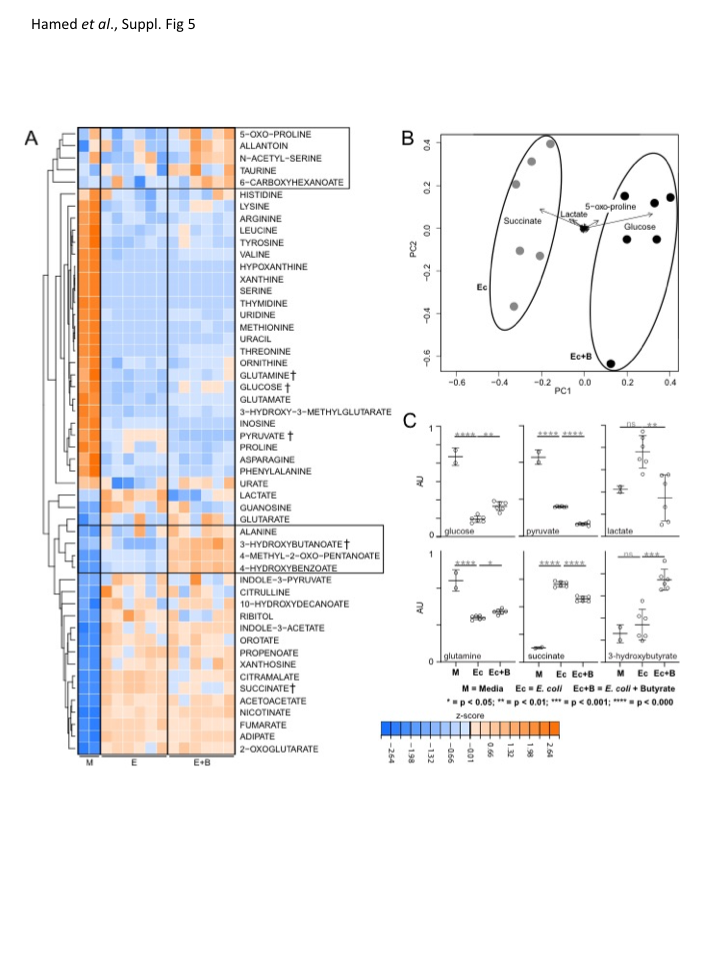

Supplement: Supplemental Material [file KGMI_A_2281011_SM8049.zip › KGMI_Supplemental figures and tables/Hamed Fig S5.tiff]

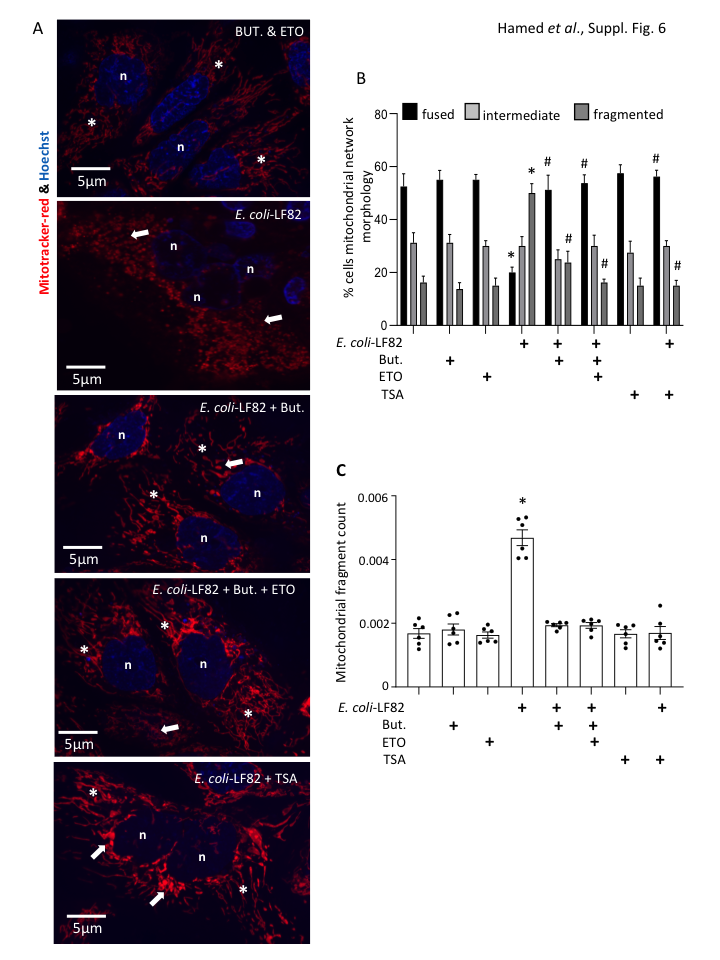

Supplement: Supplemental Material [file KGMI_A_2281011_SM8049.zip › KGMI_Supplemental figures and tables/Hamed Fig S6.tiff]

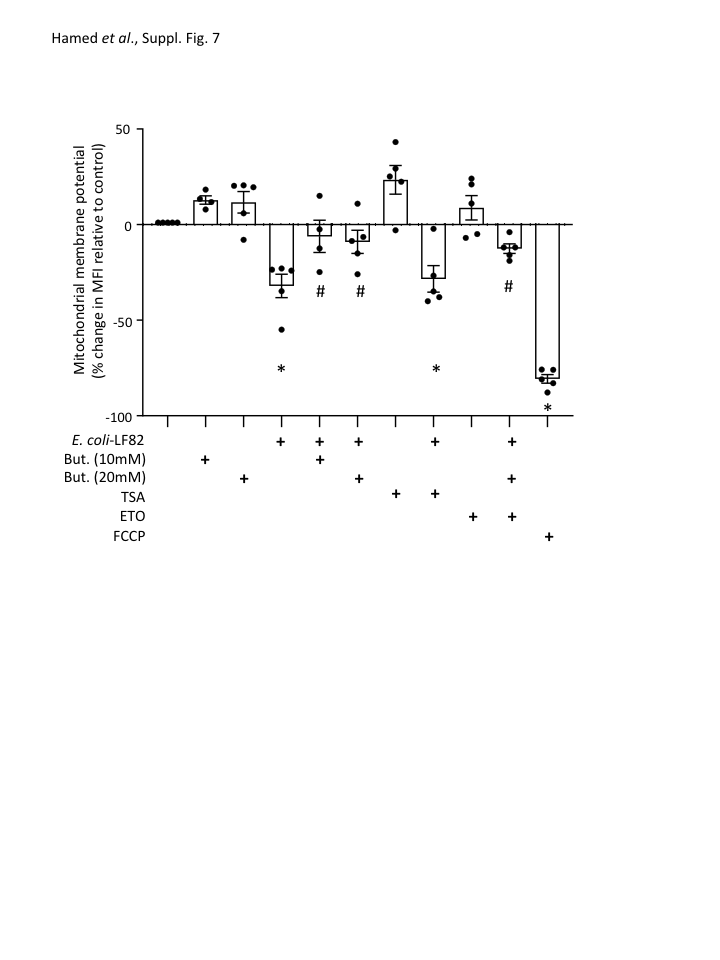

Supplement: Supplemental Material [file KGMI_A_2281011_SM8049.zip › KGMI_Supplemental figures and tables/Hamed Fig S7.tiff]

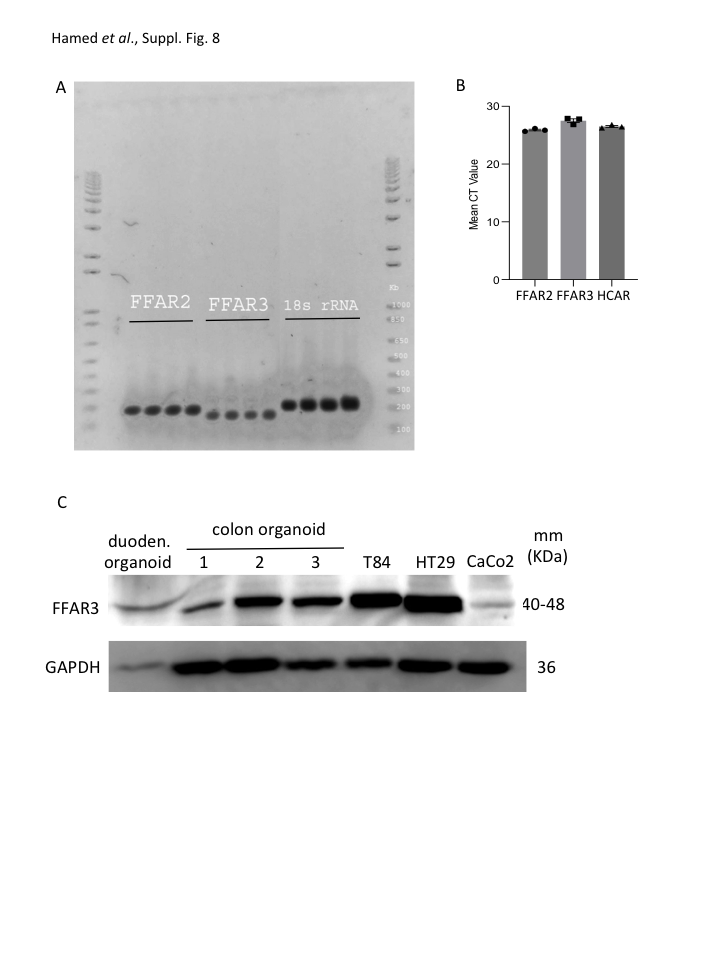

Supplement: Supplemental Material [file KGMI_A_2281011_SM8049.zip › KGMI_Supplemental figures and tables/Hamed Fig S8.tiff]
